# From Slide to Insight: The Emerging Alliance of Digital Pathology and AI in Melanoma Diagnostics

**DOI:** 10.3390/cancers17223696

**Published:** 2025-11-18

**Authors:** Federico Venturi, Giulia Veronesi, Alberto Gualandi, Elisabetta Magnaterra, Biagio Scotti, Ina Sotiri, Carlotta Baraldi, Aurora Maria Alessandrini, Leonardo Veneziano, Sabina Vaccari, Elena Maria Cama, Daniela Tassone, Barbara Corti, Emi Dika

**Affiliations:** 1 Department of Medical and Surgical Sciences (DIMEC), Alma Mater Studiorum, University of Bologna, 40138 Bologna, Italyina.sotiri@studio.unibo.it (I.S.);; 2 Oncologic Dermatology Unit, IRCCS Azienda Ospedaliero-Universitaria di Bologna, 40138 Bologna, Italy; 3 Dermatology Unit, Istituto Nazionale di Riposo e Cura per Anziani, INRCA-IRCCS Hospital, 60124 Ancona, Italy; 4 Dermatology & Venereology Department, University Hospital Center “Mother Theresa”, 11942 Tirana, Albania; 5 Plastic Surgery, IRCCS Azienda Ospedaliero-Universitaria di Bologna, 40138 Bologna, Italy; 6 Pathology Unit, IRCCS Azienda Ospedaliero-Universitaria di Bologna, 40138 Bologna, Italy; barbara.corti@aosp.bo.it

**Keywords:** cutaneous melanoma, digital pathology, artificial intelligence, whole slide imaging, convolutional neural networks, deep learning, nuclei morphology, tumor-infiltrating lymphocytes, spatial modeling, molecular histopathology

## Abstract

**Background:** Cutaneous melanoma (CM) poses significant diagnostic challenges due to its biological heterogeneity and the subjective interpretation of histopathologic criteria. While early and accurate diagnosis remains critical for patient outcomes, conventional pathology is limited by interobserver variability and diagnostic ambiguity, especially in borderline lesions. **Objective:** This narrative review explores the integration of digital pathology (DP) and artificial intelligence (AI)—including deep learning (DL), machine learning (ML), and interpretable models—into the histopathologic workflow for CM diagnosis. **Methods:** We systematically searched PubMed, Scopus, and Web of Science (2013–2025) for studies using whole slide imaging (WSI) and AI to assist melanoma diagnosis. We categorized findings across five domains: WSI-based classification models, feature extraction (e.g., mitoses, ulceration), spatial modeling and TIL analysis, molecular prediction (e.g., BRAF mutation), and interpretable pipelines based on nuclei morphology. **Results:** We included 87 studies with diverse AI methodologies. Convolutional neural networks (CNNs) achieved diagnostic accuracy comparable to expert dermatopathologists. U-Net and Mask R-CNN models enabled robust detection of critical histologic features, while nuclei-level analyses offered explainable classification strategies. Spatial and morphometric modeling allowed quantification of tumor–immune interactions, and select models inferred molecular alterations directly from H&E slides. However, generalizability remains limited due to small, homogeneous datasets and lack of external validation. **Conclusions:** AI-enhanced digital pathology holds transformative potential in CM diagnosis, offering accuracy, reproducibility, and interpretability. Yet, clinical integration requires multicentric validation, standardized protocols, and attention to workflow, ethical, and medico-legal challenges. Future developments, including multimodal AI and integration into molecular tumor boards, may redefine diagnostic precision in melanoma.

## Simple Summary

Cutaneous melanoma is a potentially lethal skin cancer that can be difficult to diagnose accurately, especially in early or ambiguous cases. Traditional histopathology relies on expert evaluation of tissue slides, but this process is subjective and prone to variability. With the rise of digital pathology and artificial intelligence (AI), there is growing interest in using computational tools to assist melanoma diagnosis. This review explores how AI—particularly deep learning and interpretable models—can analyze digital slides, extract diagnostic features, and even predict genetic mutations from routine images. By summarizing recent advances across classification, spatial modeling, and explainable AI, this work highlights how these tools can improve diagnostic accuracy, reduce workload, and support decision-making. Our goal is to inform researchers, clinicians, and pathologists of the current state of AI-assisted melanoma diagnostics and guide future studies toward more robust, clinically integrated solutions.

## 1. Introduction

Cutaneous melanoma (CM) is one of the most aggressive and biologically complex skin malignancies, accounting for a disproportionate number of skin cancer-related deaths despite representing a minority of total cases [1,2]. The global incidence of CM has been steadily increasing over the past decades, particularly among fair-skinned populations, with substantial variation in clinical behavior depending on histological subtype, anatomic location, and stage at diagnosis [3,4]. Early detection remains critical, as prognosis dramatically improves when melanoma is identified and treated at thin, localized stages [5,6,7]. However, accurate histopathological diagnosis—especially in early or atypical lesions—remains a major challenge [8]. The gold standard for melanoma diagnosis is the microscopic examination of hematoxylin and eosin (H&E)-stained tissue by dermatopathologists [9]. This assessment requires expert integration of multiple criteria, including cytological atypia, architectural disorder, mitotic rate, and the nature of tumor–stromal interactions [10,11,12,13]. However, the complexity of melanocytic lesions and the inherently subjective nature of histologic interpretation contribute to significant inter- and intra-observer variability [14]. Studies have reported diagnostic discordance rates ranging from 10% to 25% among expert pathologists, especially in difficult categories such as spitzoid lesions, nevoid melanoma, and severely dysplastic nevi [15,16]. The transition from glass slides to high-resolution whole slide images (WSIs) enables computational approaches to perform detailed quantitative assessments of tissue architecture and cellular morphology [17,18]. In parallel, advances in machine learning (ML)—particularly deep learning models such as convolutional neural networks (CNNs) and U-Net architectures—have demonstrated strong performance in cancer diagnostics, including melanoma [19,20,21,22]. These artificial intelligence (AI) systems offer multiple advantages: they can standardize diagnostic evaluation, provide real-time decision support, extract novel spatial and morphometric biomarkers, and facilitate scalable pathology services in under-resourced settings [23,24,25]. Moreover, AI is increasingly being explored not only for primary diagnosis but also for predicting genomic alterations (e.g., BRAF mutations), tumor-infiltrating lymphocytes (TIL) burden, and even treatment response [26,27,28]. Despite this promise, real-world adoption of AI in dermatopathology remains limited. Challenges include model interpretability, dataset variability, regulatory approval, integration into clinical workflows, and acceptance by pathologists. The field must also grapple with ethical and medico-legal considerations around AI-assisted diagnostics [29,30,31]. This review aims to provide a comprehensive and critical synthesis of the current role of digital pathology (DP) and AI in the histopathologic diagnosis of cutaneous melanoma. We discuss the evolution of AI applications from simple classification tasks to advanced spatial modeling and prognostic prediction. Emphasis is placed on recent developments in explainable machine learning, such as nuclei-level morphometric pipelines, and the translational implications of integrating AI into melanoma care. By highlighting current capabilities, limitations, and future directions, we aim to contextualize the growing synergy between computational tools and expert pathology in the era of precision dermatology.

## 2. Materials and Methods

This study was conducted as a structured narrative review aiming to synthesize and critically appraise the current literature on the integration of DP and AI techniques in the histopathological diagnosis of cutaneous melanoma. Emphasis was placed on studies employing WSI, machine learning (ML), and deep learning (DL) methods—including both black-box and interpretable models—targeting melanoma classification, feature extraction, molecular prediction and interpretable AI approaches including nuclei-level models.

A comprehensive literature search was performed across three major databases:

- PubMed/MEDLINE
- Scopus
- Web of Science

**Figure 1:**
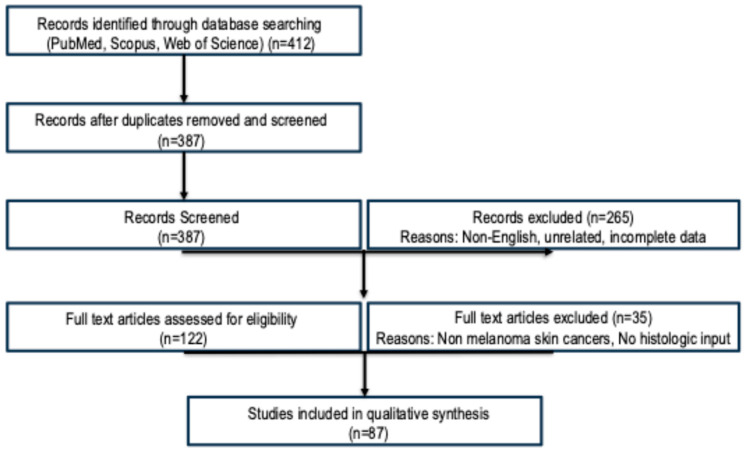
The flow diagram illustrating the study selection process for this narrative review. A total of 412 records were identified through database searches (PubMed, Scopus, Web of Science). After removal of duplicates, 387 titles and abstracts were screened. Of these, 265 were excluded based on relevance or study type. A total of 122 full-text articles were assessed for eligibility, and 35 were excluded (e.g., not focused on melanoma, lacking histologic input, or missing performance metrics). Ultimately, 87 studies met the inclusion criteria and were included in the qualitative synthesis.

The search covered the period from January 2013 to August 2025, using combinations of controlled vocabulary (MeSH terms) and free-text terms. The following Boolean search string was applied: (“melanoma” OR “cutaneous melanoma”) AND (“digital pathology” OR “whole slide imaging” OR “WSI”) AND (“artificial intelligence” OR “machine learning” OR “deep learning” OR “convolutional neural networks” OR “AI” OR “neural network” OR “U-Net” OR “pathomics” OR “computational pathology” OR “morphology” OR “spatial organization”). Studies were included if they met the following criteria: (1) original research applying AI or machine learning techniques to the histopathological diagnosis of cutaneous melanoma using digital pathology inputs; (2) used whole slide images (WSIs), image tiles, or nuclei-level segmentation derived from H&E-stained tissue; (3) reported diagnostic or predictive performance metrics (e.g., accuracy, AUC, sensitivity, specificity, F1 score); (4) were published in English in peer-reviewed journals. Reviews, editorials, case reports, and studies lacking technical or clinical validation were excluded. Following deduplication, titles and abstracts were screened for eligibility. Full texts of potentially relevant articles were then assessed independently by three reviewers (FV, GV and AG). Discrepancies were resolved by consensus. Data were extracted on study design, dataset characteristics, AI methodology (e.g., CNN, U-Net, MIL, LDA), diagnostic task (e.g., classification, segmentation, mutation prediction), performance metrics, model explainability, and external validation. Due to methodological heterogeneity, a meta-analysis was not feasible. Instead, findings were synthesized qualitatively across five conceptual domains: (1) WSI-based AI classification of melanoma; (2) feature extraction (mitoses, ulceration, Breslow thickness); (3) spatial modeling and TIL analysis; (4) molecular prediction using AI; (5) interpretable and nuclei-level AI approaches. Emphasis was placed on clinical relevance, reproducibility, generalizability, and translational potential. Limitations including dataset bias, lack of standardization, and integration challenges were also noted. Where applicable, recent systematic reviews and meta-analyses were used to support pooled sensitivity/specificity data. No ethics approval was required for this literature-based study (Figure 1).

## 3. Results

A total of 87 studies published between January 2013 and August 2025 met the inclusion criteria and were analyzed across five key domains: WSI-based classification models, histologic feature extraction, spatial modeling including TIL quantification, molecular prediction, and interpretable AI approaches focused on nuclei-level analysis. Collectively, these studies illustrate a rapidly evolving field at the intersection of computational pathology and melanoma diagnostics. The integration of DP and AI in the histopathological diagnosis of cutaneous melanoma has advanced rapidly, with a particular emphasis on WSI, ML, and DL approaches. WSI enables high-resolution digitization of entire histopathology slides, facilitating computational analysis and remote review. DL, especially CNNs, has demonstrated high diagnostic accuracy for melanoma classification, often matching or exceeding human pathologists in sensitivity and specificity, though variability in image acquisition and annotation remains a challenge [17,31,32,33,34,35,36]. Studies employing both “black-box” and interpretable models have shown that automated algorithms can reliably distinguish melanoma from benign melanocytic lesions, with pooled sensitivity and specificity approaching 90% and 92%, respectively, in meta-analyses [17,33,35]. Feature extraction using DL models has enabled identification of histopathological patterns and prognostic markers, while emerging work in molecular prediction leverages image-based surrogates for genetic and mutational status, though these applications remain investigational [34,35,37,38].

### 3.1. WSI-Based AI Classification of Melanoma

WSI has enabled large-scale digitalization of histopathological slides, providing a foundation for the application of AI-based classification systems [39]. Particularly, WSI-based artificial intelligence AI for melanoma classification has rapidly advanced [34,35,37,38], with multiple systematic reviews and meta-analyses demonstrating high diagnostic accuracy. Recent meta-analyses report pooled sensitivities and specificities for automated image analysis algorithms applied to melanoma histology in the range of 89–92% and 90–94%, respectively, with area under the curve (AUC) values up to 0.96–0.98 for hybrid and deep learning models [33,40,41]. Hekler et al. developed a deep CNN that classified melanoma vs. nevus with an AUC > 0.94 [42]. Large reader studies confirm that CNNs outperform the majority of dermatologists in both sensitivity and specificity, with CNNs achieving sensitivities of 82–87% and specificities of 77–86%, compared to dermatologists sensitivities of 67–89% and specificities of 60–75% [22,43]. For histopathological melanoma diagnosis, CNNs have demonstrated higher accuracy than panels of expert pathologists, with CNNs achieving 68% accuracy versus 59% for pathologists in challenging image-based tasks [44]. However, real-world reproducibility and accuracy among pathologists remain variable, with consensus panel accuracy for invasive melanoma at 72–82% and lower for early-stage or ambiguous lesions [45]. Attention-based pooling strategies and multi-instance learning (MIL) frameworks further improved diagnostic localization, enabling the models to focus on the most histologically relevant regions [18,46]. These results suggest that AI models, particularly CNNs and hybrid approaches, can match or exceed the diagnostic performance of clinicians in controlled settings. WSI-based AI models have shown particular promise in distinguishing melanoma from benign melanocytic lesions, with some studies reporting superior or at least equivalent performance compared to experienced dermatopathologists [33,36,47]. However, most studies are limited by small, homogeneous datasets, lack of external validation, and artificial test settings that do not fully represent real-world clinical diversity or workflow [17,32,47]. Recent multicenter initiatives have begun to address this issue by applying federated learning strategies and cross-institutional datasets that preserve patient privacy while enhancing model generalizability. Consensus is emerging that external validation should include: (i) at least one independent dataset from a different institution; (ii) inclusion of rare subtypes and variable histologic patterns; and (iii) evaluation across diverse patient populations, including different skin phototypes and age groups. Studies failing to meet these criteria may risk overfitting and limited translational relevance.

Variability in image acquisition, pre-processing, and annotation standards further limits generalizability and reproducibility [17,32]. For instance, Maron et al. demonstrated that CNN performance dropped substantially when exposed to out-of-distribution (OOD) data, such as images with artificial corruptions (e.g., blur, noise, brightness changes) or minor perturbations (e.g., small rotations, zooms). In their benchmark, the mean accuracy of four CNN architectures decreased from 85 to 88% on unmodified images to as low as 65–70% on corrupted or perturbed images, representing a performance drop of up to 23 percentage points due to image quality variability [48]. Similarly, Maron et al. found that even minor, clinically irrelevant changes in image acquisition (e.g., different angles, lighting, or zoom) led to inconsistent predictions, with CNNs showing “brittleness”—the probability of a model changing its diagnosis for the same lesion across different images ranged from 10% to 30% [49]. Schmitt et al. quantified the impact of hidden batch effects in digital pathology: CNNs could learn non-biological variables such as slide origin or scanner type, achieving up to 100% accuracy in distinguishing slides by origin, which can confound diagnostic predictions and reduce generalizability [50]. Cho et al. further showed that CNNs trained on high-quality, standardized images performed well, but their accuracy dropped markedly on unstandardized, out-of-focus, or poorly lit clinical photographs, with performance reductions of 10–20 percentage points in AUC or sensitivity [51]. These findings underscore that CNN diagnostic accuracy for melanoma can decrease by 10–23 percentage points or more when exposed to variability in image quality, acquisition parameters, or hidden batch effects, highlighting the need for robust model development and diverse, well-curated datasets [48,49,50,51]. Despite these limitations, AI-assisted WSI analysis is recognized as a valuable adjunct to pathologists, improving workflow efficiency and providing a reliable second opinion, especially for less experienced clinicians. However, current consensus is that these tools should not replace expert histopathological assessment but rather serve as decision support, pending further validation in diverse, prospective clinical cohorts and standardization of methodologies [17,32,33]. Key challenges remain in model explainability, integration into clinical workflows, and ensuring robust performance across populations and melanoma subtypes. Commonly reported limitations or biases in studies using whole slide imaging (WSI) for the classification of melanoma include the interobserver variability and reference standard bias. There is substantial discordance among pathologists in classifying melanocytic lesions, with reported rates up to 25%, which complicates the establishment of a reliable ground truth for AI training and validation. This variability can introduce bias in both the development and assessment of WSI-based AI models [52]. Moreover, WSI-based interpretation is less accurate for certain lesion classes, especially intermediate or ambiguous lesions (e.g., class III lesions), leading to higher rates of discordance and potential misclassification between benign and malignant categories [53]. A further critical limitation is the lack of sufficient, high-quality annotated datasets for rare or diagnostically challenging melanoma subtypes, such as desmoplastic melanoma, Spitzoid lesions, and other tumors within the biologic gray zone. These subtypes are both uncommon and marked by substantial interobserver variability, making consistent ground truth labeling difficult. As a result, most existing AI models are trained predominantly on common melanocytic lesions and may perform suboptimally on rare or ambiguous cases—precisely those that most require diagnostic support. This underrepresentation substantially limits robustness, external validity, and real-world applicability, highlighting the urgent need for larger, multicenter, pathologist-curated datasets that adequately capture the full histopathological diversity of melanoma. Results are displayed and integrated in Table 1.

### 3.2. Feature Extraction: Mitoses, Ulceration, and Tumor Thickness

Several studies used semantic segmentation and object detection techniques to extract key histopathological features such as mitotic figures, ulceration, and Breslow thickness—parameters critical for melanoma staging and prognosis [54,55]. U-Net and Mask R-CNN architectures were commonly applied, demonstrating strong performance in identifying mitoses, delineating the epidermal–dermal junction, and quantifying ulcerated regions [56]. Automated Breslow thickness estimation showed excellent correlation with pathologist assessments in multiple studies, suggesting its potential utility in quality control and standardization [54,57]. These features are critical for diagnosis and prognostication, as emphasized in the clinical literature [58]. Automated detection of mitotic figures remains challenging due to their rarity and morphological variability. Deep learning models, particularly convolutional neural networks (CNNs), have shown promise in identifying mitotic figures, but performance is still inferior to expert pathologists in complex cases. Studies highlight the need for larger annotated datasets and improved interpretability to enhance reliability [34,35]. Feature extraction pipelines often use nuclei segmentation and morphological analysis to approximate mitotic activity, but validation against manual counts is essential [34,59]. AI-based approaches for ulceration detection typically rely on segmentation algorithms to delineate the epidermal surface and identify areas of tissue disruption. While some models achieve high sensitivity and specificity, ulceration remains a less frequently targeted feature in current AI literature compared to tumor thickness and mitoses. Integration of clinical metadata and multimodal imaging may improve accuracy [34,35,60]. Automated measurement of Breslow thickness is a major focus, given its prognostic significance. Deep learning models can segment tumor boundaries and estimate thickness with high concordance to manual assessment, though challenges persist with poorly demarcated lesions and artifacts [33,34,35]. Recent systematic reviews report pooled sensitivities and specificities above 90% for automated image analysis in melanoma histology, but emphasize heterogeneity and the need for further validation before clinical adoption [33,35]. Overall, AI and digital pathology offer high accuracy and reproducibility for feature extraction in melanoma, but limitations include interobserver variability, data heterogeneity, and lack of standardized protocols. Continued collaboration between pathologists and computer scientists is essential to address these challenges and facilitate clinical integration [31,35,61]. Results are displayed and integrated in Table 1.

### 3.3. Spatial Modeling and Tumor-Infiltrating Lymphocyte (TIL) Analysis

The integration of AI with spatial histopathology enables quantification of tumor architecture and the immune microenvironment [59]. Several studies leveraged deep learning to detect and spatially map TILs across melanoma sections with improved reproducibility, objectivity, and prognostic value compared to traditional manual assessment. Manual TIL scoring, typically performed by pathologists using H&E-stained slides, suffers from significant interobserver variability and limited consensus on grading systems, which restricts its prognostic utility in melanoma [62,63,64,65]. Models using CNNs and graph-based representations were able to identify immune cell clusters and their proximity to tumor nests—features increasingly recognized as predictive of immunotherapy response. Moore et al. demonstrated that AI-derived TIL spatial patterns correlated with transcriptomic immune signatures, while other groups showed associations with progression-free survival and overall survival in treated patients [27,64]. Spatial modeling frameworks, including those integrating histopathology with transcriptomics, enable quantitative characterization of the TME, revealing spatial cellular architectures and immune infiltration patterns that are not apparent from molecular data alone. These spatial features can distinguish microenvironment subtypes and predict patient prognosis, enhancing the interpretability and clinical relevance of computational histopathology [66]. Additionally, machine learning models leveraging nuclei morphology and spatial organization have shown promise in automated melanoma detection, providing interpretable results that align with established histopathological criteria and supporting clinical decision-making [59]. Systematic reviews confirm that deep learning and image analysis algorithms achieve high diagnostic accuracy (mean sensitivity and specificity >90%) in melanoma histopathology, though further validation and standardization are needed for clinical integration [17,33,34,35]. Multiplexed immunohistochemistry and digital pathology preserve spatial context and enable in situ single-cell profiling, facilitating detailed study of cell–cell interactions and tissue architecture relevant to immunotherapy response [67]. Emerging 3D histology models may further enhance spatial assessment, though technical limitations remain [68]. Results are displayed and integrated in Table 1.

**Table 1:** Diagnostic performance of AI models across key histopathological tasks in melanoma.

Task	Model Types	Accuracy (Range)	AUC (Range)	No. of Studies
Melanoma Classification	CNN, MIL	0.85–0.96	0.91–0.98	18
Mitosis Detection	U-Net, Mask R-CNN	0.78–0.88	0.83–0.89	11
Breslow Thickness Estimation	CNN, Regression	0.86–0.92	0.90–0.95	9
Ulceration Detection	Segmentation	0.78–0.85	0.80–0.88	7
TIL Analysis	Graph CNN, Spatial Maps	0.84–0.90	0.88–0.93	10
Molecular Prediction	CNN, Multimodal Fusion	0.75–0.82	0.80–0.86	6

This table synthesizes representative performance ranges reported in studies published between 2013 and 2025: melanoma classification, mitosis detection, Breslow thickness estimation, ulceration detection, tumor-infiltrating lymphocyte (TIL) analysis, and molecular prediction. Data synthesized from 61 studies published between 2013 and 2025. Convolutional neural networks (CNNs), U-Net, Mask R-CNN, and hybrid deep learning approaches were the most frequently used architectures. Performance metrics reflect retrospective, mostly single-institution cohorts.

### 3.4. Molecular Prediction from Histopathology

Emerging studies explored the use of AI to predict genomic alterations directly from digitized histologic slides. Emerging studies show that DL models can infer molecular features such as BRAF mutation status and other actionable genomic alterations directly from routine hematoxylin and eosin (H&E) slides, although these applications remain investigational and require further validation before clinical integration [69,70]. Multiplexed immunohistochemistry combined with digital pathology enables spatial mapping of the tumor microenvironment, supporting personalized therapy decisions and molecular characterization [67,71]. Coudray et al. first demonstrated genomic prediction across cancers; subsequent melanoma specific works achieved 75–85% accuracy for BRAF status [72,73,74,75,76]. Morphological surrogates (e.g., pagetoid spread, cytologic atypia, and architectural patterns) were hypothesized as mediating features. More recent models trained on pan-cancer datasets have expanded their scope to predict transcriptomic programs such as immune evasion, angiogenesis, or MAPK pathway activation [77]. These findings support the growing interest in “molecular histopathology”—the use of AI to infer biologic phenotype from morphology alone. Nevertheless, performance remains inferior to gold-standard molecular techniques, and these models currently serve as adjunctive screening tools rather than replacements. The literature emphasizes the need for high-quality, diverse datasets and collaboration between pathologists, computer scientists, and bioinformaticians to address these challenges and ensure safe, effective translation of AI tools into routine practice [31,61]. Results are displayed and integrated in Table 1.

### 3.5. Interpretable AI and Nuclei-Level Feature Models

A key advancement in recent years has been the development of interpretable machine learning pipelines that mirror the reasoning of trained pathologists. These models focus on extracting quantifiable nuclear morphologic and spatial features from whole-slide images, emulating the cellular-level assessment performed by pathologists. For example, machine learning approaches that segment nuclei and synthesize geometric, morphologic, and spatial variables have shown robust performance in distinguishing melanoma from nevi, with interpretability grounded in established histopathological criteria. Such models enable clinicians to verify and understand the diagnostic process, supporting clinical decision-making and prioritization of complex cases [59].One notable example is the work by Veronesi et al., who proposed a method combining U-Net-based nuclear segmentation with linear discriminant analysis (LDA) on spatial and morphologic features [59]. Their pipeline extracted over 6 million nuclei from WSIs, quantifying 44 nuclear and spatial variables, including area, eccentricity, clustering, and local heterogeneity. The model achieved an accuracy of 90.4%, with a sensitivity of 84.4% and precision of 86.5%, while maintaining full interpretability at the feature level. Importantly, variables such as nuclear pleomorphism, spacing irregularity, and anisotropy—long appreciated by expert dermatopathologists—were among the strongest discriminators between melanoma and nevi. Systematic reviews confirm that deep learning and machine learning algorithms applied to digital histopathology images of melanoma achieve high diagnostic accuracy, with pooled sensitivity and specificity approaching 90% and 92%, respectively. However, these studies highlight heterogeneity in methodology, limited external validation, and challenges in generalizability across diverse populations and image acquisition protocols [32,34].

### 3.6. Molecular Tumor Board and AI in Melanoma

Alongside diagnostic applications, AI is increasingly being integrated into clinical decision-making frameworks, particularly within the Molecular Tumor Board (MTB), which represents a cornerstone of precision oncology in advanced melanoma. The MTB enables the multidisciplinary integration of clinical, pathological, and molecular data to support personalized therapeutic strategies [78]. The growing adoption of next-generation sequencing (NGS) has expanded both the diagnostic potential and the interpretative complexity of molecular results, often exceeding the capacity of conventional decision-making processes [79]. In this context, AI can substantially enhance MTB activities through different algorithmic approaches. Machine learning and deep learning models can automate mutation detection from sequencing data, predict their functional relevance, and assist in classifying variants of uncertain significance (VUS) by integrating molecular, clinical, and histopathological parameters [80]. Supervised algorithms and deep neural networks are already employed to discriminate driver from passenger mutations, assessing their impact on signaling pathways and their potential therapeutic value [81,82]. In parallel, semi-supervised learning and clustering models can group variants with similar molecular profiles, facilitating automated interpretation [83]. A further step forward is represented by multimodal models, capable of correlating genomic and transcriptomic data with clinical, histopathological, and radiological information to generate therapy-response predictions and identify candidates for clinical trials [84]. An additional and emerging field where the MTB–AI alliance may provide substantial benefit concerns the management of melanocytic lesions within the biologic gray zone, encompassing melanocytomas, MELTUMPs, and other non-conventional melanocytic tumors [85,86]. These entities, which exhibit overlapping morphologic, molecular, and biological features between benign nevi and overt melanomas, remain a major source of diagnostic uncertainty and therapeutic ambiguity. Here, AI-driven integrative analytics could play a transformative role by learning latent molecular–morphologic signatures that escape human perception and correlating them with clinical outcomes. Such approaches could help standardize risk stratification and improve reproducibility across experts, supporting the MTB in distinguishing lesions with intermediate biological potential, such as atypical Spitz tumors or BAP1-inactivated melanocytic tumors, from true melanomas [15,87]. By leveraging explainable AI and multimodal fusion of dermoscopic, histologic, genomic, and clinical data, MTBs may move toward a data-informed and biologically grounded redefinition of the gray zone, bridging diagnostic uncertainty and therapeutic precision [88,89]. However, the adoption of AI within MTBs requires rigorous clinical validation, algorithmic transparency, and continuous monitoring, as clinical safety and trust remain central to decision-making [88]. To be acceptable in clinical practice, AI models must incorporate interpretable components and always leave the final decision to the clinician. The use of explainability tools or feature contribution analyses, helps clarify why an algorithm suggests a given strategy or variant interpretation [90,91].

### 3.7. Workflow Integration, Accessibility, and Open-Source Tools

While technical performance is promising, real-world integration of AI into histopathologic workflows remains limited. Workflow integration remains a key challenge. Most AI systems are still investigational, with limited real-world deployment due to issues such as robustness across diverse datasets, lack of external validation, and the need for seamless interoperability with laboratory information systems [92,93]. Effective integration requires collaboration between pathologists, computer scientists, and engineers, as well as standardized reporting and validation protocols. Accessibility is improving with the proliferation of public datasets (e.g., ISIC, HAM10000), but generalizability is limited by underrepresentation of skin of color and non-Western populations [32,92].

Beyond technical validation, regulatory approval represents a major barrier to clinical implementation. In most jurisdictions, AI models intended for diagnostic use must undergo rigorous review to obtain regulatory clearance. However, the absence of standardized evaluation frameworks and the complexity of AI behavior (e.g., adaptive algorithms, black-box models) have made these pathways uncertain and often prohibitively time-consuming. In parallel, logistical bottlenecks persist. Many pathology labs—particularly in community or non-academic settings—lack access to WSI scanners, high-performance computational infrastructure, and IT support required for real-time inference and integration with digital workflow systems. The costs of digitization, data storage, and personnel training further complicate large-scale adoption. As such, AI solutions remain largely confined to research institutions and early-adopter centers. Addressing these barriers will require dedicated funding, collaborative infrastructure initiatives, and regulatory innovation.

Open-source tools are increasingly available, exemplified by the development of 3D histology models using open-source software, which enhance anatomical assessment and may enter routine practice within the next decade. However, computational limitations and data privacy concerns persist. In addition to these challenges, widespread implementation of AI-assisted digital pathology is hindered by the substantial infrastructure required to support high-throughput workflows. These include high-resolution WSI scanners, dedicated servers or cloud-based high-performance computing resources for model training and inference, and scalable storage solutions capable of managing terabyte-scale slide repositories. Such systems also require robust network bandwidth, integration with laboratory information systems (LIS), and ongoing technical support. These infrastructural demands represent a significant financial and logistical barrier for many institutions—particularly smaller hospitals, community practices, and centers in low-resource settings—thereby limiting equitable adoption of AI technologies in melanoma diagnostics. The latest evidence demonstrated that artificial intelligence models for melanoma histopathology achieve high diagnostic accuracy in external validation studies, but generalizability across diverse populations and real-world clinical settings remains limited. Recent multicenter studies using federated learning approaches have shown robust external performance, with AUROC values exceeding 0.91 on out-of-distribution datasets that include a broad spectrum of melanoma subtypes, anatomical sites, and patient ages. These models, trained prospectively across multiple institutions, effectively minimize selection bias and better reflect real-world clinical heterogeneity, supporting their reliability for routine diagnostic use [52]. However, most datasets are still derived from European and North American populations, and representation of skin of color (Fitzpatrick IV-VI) and non-Western populations is insufficient, limiting the applicability of these models globally [32,94]. For deep learning models to be effectively implemented in clinical practice, they must be trained on datasets from diverse sources. The greater the variety of data a model is exposed to, the better it can generalize and accurately predict outcomes on new, unseen cases. Meta-analyses and umbrella reviews confirm that deep learning and hybrid models consistently outperform or match experienced dermatologists in diagnostic accuracy, with pooled sensitivities and specificities around 89–92% [40,41]. Nevertheless, studies highlight that external validation is infrequent, and performance may decrease when models are applied to populations or image sources not represented in the training data [32,92,94]. There is a critical need for standardized reporting, inclusion of diverse patient cohorts, and transparent benchmarking to ensure equitable and effective deployment in clinical practice. Despite promising results, the reproducibility and generalizability of AI models remain major challenges in melanoma histopathology. A core limitation is the lack of standardized validation metrics and benchmarking protocols across studies. Many works report only internal performance without external testing, use inconsistent definitions of sensitivity or accuracy, or lack calibration metrics such as confidence intervals and error margins. This heterogeneity makes direct comparison difficult and limits the establishment of performance baselines for clinical translation. Moreover, published models often underperform when evaluated on out-of-distribution datasets, highlighting their limited robustness to variations in staining, scanner type, or population demographics. To address this, expert consensus on core outcome metrics, minimum dataset diversity requirements, and independent external validation should be prioritized. Without such harmonization, clinical implementation will remain fragmented and prone to error. Furthermore, a key challenge with AI is that its outputs often do not intuitively align with traditional clinical reasoning or human logic. These models frequently function as “black boxes”, generating results without providing clear explanations for how specific conclusions are reached. This lack of interpretability undermines trust in AI systems and poses a significant barrier to their adoption in clinical practice. Another critical human factor challenge in adopting AI-assisted melanoma diagnostics is automation bias—the tendency of clinicians to overly rely on algorithmic outputs, even in the face of conflicting clinical or pathological data. This is particularly problematic in the evaluation of complex melanocytic lesions, such as atypical Spitz tumors, desmoplastic melanomas, or MELTUMPs, where nuanced interpretation and clinical-pathological correlation remain essential. Over-reliance on AI in these cases can lead to diagnostic complacency and potentially harmful misclassification. To prevent this, AI systems should be framed as decision-support tools, not diagnostic authorities, and their outputs should always be reviewed within the broader clinical context. Human-in-the-loop models, interpretability features, and rigorous validation in edge-case subtypes are necessary to reduce automation bias and reinforce the primacy of expert judgment in final diagnosis.

### 3.8. Workload, Time and Resources

Pathology has become a discipline where time, expertise, and technological demand converge, shaping not only diagnostic accuracy but also the rhythm and sustainability of clinical work. Time is both a technical variable and a critical factor. Ancillary investigations, such as immunohistochemistry (IHC) in doubtful cases or molecular analysis in complex ones, remain needful but are also demanding in terms of tissue use and time, inevitably leading to costs and delays [95]. They require expertise and a delicate balance between accuracy and sustainability, often becoming an organizational bottleneck [96].

The spread of WSI has expanded diagnostic possibilities but also the volume and complexity of image analysis [97], increasing cognitive load and the risk of omission. Digitalization, while improving storage and sharing, also introduces new forms of visual fatigue and distraction [98]. This scenario is intertwined with the chronic shortage of laboratory staff and reveals a paradox: pathology is increasingly central to precision medicine, yet threatened by overload and fatigue, with an increased risk of error [99]. In this context, digital and AI tools in dermatopathological diagnosis are not merely aids to efficiency but genuine “cognitive offloading devices” [100], automating repetitive yet sophisticated tasks and freeing the pathologist’s mental resources for interpretation and critical judgment [98]. AI is destined to become an integral part of pathology and if accompanied by robust validation, clear rules and attention to human factors, not only contributes to efficiency, but also to a real improvement in cognitive well-being, reducing the risk of errors linked to mental fatigue and overload [101]. In this context, AI applications function as cognitive offloading tools—systems that absorb repetitive, high-volume, or visually taxing tasks such as mitotic figure identification, ulceration segmentation, or nuclei counting—thereby freeing the pathologist’s mental bandwidth for complex diagnostic reasoning. This redistribution of cognitive effort can significantly improve diagnostic quality and reduce burnout, particularly in high-throughput academic or cancer center settings. However, it also raises ethical questions regarding task delegation, responsibility for diagnostic error, and the evolving scope of the pathologist’s expertise. Moreover, effective cognitive offloading requires trust in the system’s reliability and transparency. If AI tools are perceived as black boxes or provide inconsistent outputs, they may paradoxically increase cognitive burden by demanding additional verification steps. To mitigate this, AI systems must be explainable, auditable, and seamlessly integrated into diagnostic workflows, supporting rather than supplanting clinical judgment. Human–AI collaboration should be designed to maximize complementarity—leveraging the pattern recognition capacity of algorithms alongside the contextual insight and clinical accountability of the pathologist.

**Figure 2:**
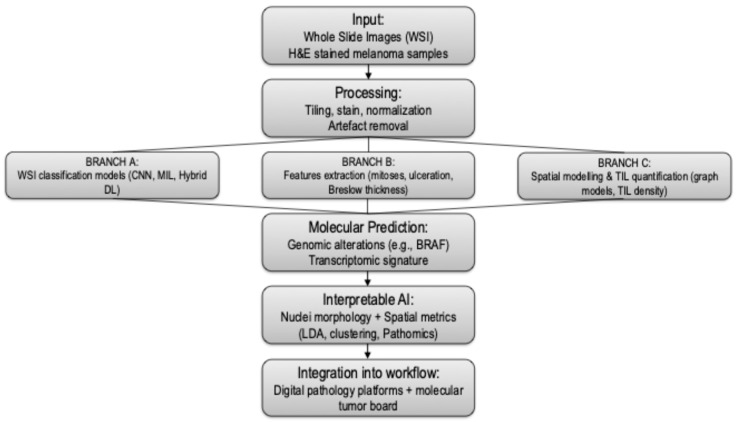
Comprehensive AI workflow for histopathologic melanoma diagnosis.

Ultimately, costs, time and resources are not just organizational variables, but reflections of the contemporary tension between scarcity of resources and data richness, calling for a renewed balance between human expertise and automation (Figure 2).

This schematic outlines the integration of digital pathology (DP) and artificial intelligence (AI) in the diagnostic workflow for cutaneous melanoma. The pipeline begins with whole slide imaging (WSI), where histopathologic slides are digitized at high resolution. Preprocessing steps include tiling, stain normalization, and quality control. Image tiles are then analyzed using convolutional neural networks (CNNs) and U-Net architectures for classification tasks (e.g., melanoma vs. nevus), semantic segmentation (e.g., Breslow thickness, ulceration), and feature extraction (e.g., mitotic figures).

Advanced modules incorporate spatial modeling of tumor-infiltrating lymphocytes (TILs) and nuclei-level morphometric analysis, providing interpretable insights into tumor architecture and immune contexture. Molecular prediction layers aim to infer genomic alterations (e.g., BRAF, NRAS mutations) and transcriptomic phenotypes directly from H&E slides. Outputs are synthesized into diagnostic and prognostic scores and can be integrated with clinical data in decision-support platforms and Molecular Tumor Boards (MTBs).

This workflow supports standardized diagnosis, risk stratification, and precision oncology approaches, while highlighting the importance of explainable AI, robust validation, and ethical integration into real-world pathology practice.

## 4. Discussion and Conclusions

The integration of AI and DP into the histopathologic diagnosis of cutaneous melanoma represents one of the most significant shifts in dermatopathology over the past decade. This review synthesizes evidence across five key domains—WSI-based classification, histopathologic feature extraction, spatial modeling, molecular prediction, and interpretable AI pipelines—highlighting the evolving role of computational tools in melanoma diagnostics. Our findings confirm that deep learning models, particularly CNNs, achieve diagnostic accuracy that is comparable to expert dermatopathologists, especially in differentiating melanoma from benign melanocytic lesions. However, despite high performance in retrospective datasets, most AI systems have not yet been prospectively validated in real-world, multi-institutional settings. Feature extraction tasks such as automated measurement of Breslow thickness, mitotic count, and ulceration detection demonstrate high promise for standardizing staging parameters and reducing interobserver variability. However, performance varies significantly depending on lesion quality, image resolution, and annotation fidelity. Spatial modeling, particularly for TILs, is emerging as a powerful tool to decode the tumor microenvironment and potentially predict immunotherapy outcomes. Furthermore, recent efforts to link histomorphology with underlying genomic alterations, such as BRAF mutation status or MAPK pathway activity, represent a key step toward “molecular histopathology”. Although still investigational, these AI-powered inferences from H&E images could one day serve as triage tools or complements to genomic sequencing. In parallel, the role of AI is expanding beyond diagnosis into clinical decision-making, particularly through its incorporation into MTBs. AI can assist in interpreting complex genomic variants, suggest targeted therapies, and even help stratify melanocytic lesions of uncertain malignant potential. This is especially valuable in the biologic gray zone of melanocytomas, MELTUMPs, and other borderline lesions where traditional criteria fail to provide diagnostic or therapeutic clarity. Multimodal AI systems that integrate dermoscopy, histopathology, molecular data, and clinical outcomes could redefine diagnostic paradigms and bring biologic meaning to morphologic ambiguity. Despite promising results, limitations persist: model generalizability across diverse populations and slide preparation protocols is not assured, and the opacity of many DL models hinders clinical trust and regulatory approval [31,32,35]. Types of technical variability include discrepancies in scanner resolution and image compression formats, variation in H&E staining intensity and hue, differences in tissue section thickness, and batch effects from slide preparation workflows. Human variability stems from pathologist annotation style, differing diagnostic thresholds for ambiguous melanocytic lesions, and inconsistent ROI marking across institutions. These factors affect the consistency of the “ground truth” and the reproducibility of model training and validation outcomes [48,49,50,51]. Interpretable models and explainable AI are increasingly emphasized to address these concerns, but robust external validation and standardized datasets are needed for clinical translation. This challenge has led to increasing demand for external validation frameworks that go beyond internal cross-validation. Robust external validation is now seen as essential for clinical adoption and should include geographically and demographically diverse datasets, inclusion of diagnostically ambiguous subtypes, and benchmarking against expert performance. Federated learning, ensemble models trained on multicenter data, and public challenges (e.g., CAMELYON, MIDOG) are helping shape consensus standards for minimal validation requirements. Without these, even high-performance models risk failing under real-world variability. Current consensus is that AI serves as a valuable adjunct to expert pathology, improving workflow efficiency and diagnostic reproducibility, but is not a replacement for human expertise [36,93,102,103]. Moreover, real-world deployment of AI-assisted digital pathology is constrained by non-trivial infrastructural requirements. The acquisition of WSI scanners, high-performance computing hardware, secure large-scale data storage, and interoperable digital pathology platforms demands substantial institutional investment. These barriers disproportionately affect smaller or non-academic centers, creating disparities in access to advanced computational diagnostics. As a result, even high-performing AI models may remain confined to well-resourced institutions unless cost-effective, scalable infrastructure solutions become more widely available.

## Abbreviations

The following abbreviations are used in this manuscript:

CM	Cutaneous melanoma
WSI	whole slide images
ML	machine learning
CNN	convolutional neural network
AI	artificial intelligence
TILs	tumor-infiltrating lymphocytes
DP	digital pathology
AUC	area under the curve
H&E	hematoxylin and eosin
LDA	linear discriminant analysis
MTB	Molecular Tumor Board
NGS	next-generation sequencing
